# Psychometric Evaluation of the 15-Item Five Facet Mindfulness Questionnaire: A Cross-Cultural Comparison Study Among English- and Chinese-Speaking Adult Mental Health Service Users

**DOI:** 10.3390/healthcare14030307

**Published:** 2026-01-26

**Authors:** Ming Yu Claudia Wong, Guangzhe Frank Yuan, Shan-yan Huang, Amos En Zhe Lian, Görkem Derin, Aslı Dila Akiş, Peejay D. Bengwasan, Hong Wang Fung

**Affiliations:** 1 Department of Health and Physical Education, The Education University of Hong Kong, Tai Po, Hong Kong, China; 2 School of Education Science, Leshan Normal University, Leshan 614000, China; yb97316@umac.mo; 3 Department of Marketing and Supply Chain Management, Overseas Chinese University, Taichung City 407, Taiwan; hsy1118@ocu.edu.tw; 4 Faculty of Social Sciences, Raffles University, Johor Bahru 79250, Malaysia; 5 Kepha Institute, Columbia International University, Columbia, SC 29203, USA; amoslianenzhe@raffles-university.edu.my; 6 Psychotraumatology and Psychohistory Research Unit, Department of Social Sciences, Institute of Forensic Sciences and Legal Medicine, Istanbul University-Cerrahpaşa, Istanbul 34320, Turkey; gorkem.derin@gmail.com; 7 Department of Psychology, Haliç University, İstanbul 34060, Turkey; aslidilaakis@halic.edu.tr; 8 Department of Psychology, De La Salle University, Taft, Manila 1004, Philippines; peejay.bengwasan@dlsu.edu.ph; 9 School of Nursing, The Hong Kong Polytechnic University, Kowloon, Hong Kong, China; 10 Mental Health Research Centre, The Hong Kong Polytechnic University, Kowloon, Hong Kong, China

**Keywords:** mindfulness, psychometrics, measurement invariance, FFMQ-15, Chinese-English comparison, cross-cultural validation, mental health service users

## Abstract

**Objectives**: Mindfulness has been proposed as an important health outcome and an indicator of mental well-being. This study aimed to evaluate the psychometric properties of the Five Facet Mindfulness Questionnaire (FFMQ-15) in two samples of mental health service users with diverse cultural and linguistic backgrounds (English- and Chinese-speaking). The study addresses the conceptual gap regarding the limited validation of the FFMQ-15 in Chinese-speaking clinical populations and examines the implications of measurement invariance. This study aimed at (1) confirming the reliability and validity of the FFMQ-15 in mental health service users; (2) assessing the validity of the FFMQ-15 in Chinese-speaking populations, where evidence is limited; and (3) examining measurement invariance across English- and Chinese-speaking groups to ensure cross-cultural applicability and comparable score interpretation. **Methods**: Participants were recruited using snowball sampling and social media advertising, targeting adults aged 18 or older who could read and write English or Chinese and had received mental health services. The English-speaking sample comprised 115 adults, and the Chinese-speaking sample included 118 adults. Exploratory factor analysis was used to identify structural dimensions, while confirmatory factor analysis was conducted for both samples to evaluate the five-factor structure of the FFMQ-15. **Results**: The EFA showed literature-aligned results supporting the 5-factor structure model, while the CFA model demonstrated acceptable fit: χ^2^/df = 159.50/80 = 1.99, *p* < 0.001; CFI = 0.927; TLI = 0.904; RMSEA = 0.065 (90% CI [0.050, 0.080]); SRMR = 0.060, BIC = 10,843.067, meeting established thresholds, and the non-significant measurement variance indicated the measurement model’s consistency among clinical patients and across different cultural contexts. **Conclusions**: The FFMQ-15 shows strong psychometric properties for measuring mindfulness in English- and Chinese-speaking mental health service users, supporting its value in clinical research and practice.

## 1. Introduction

Mindfulness has been proposed as an important health outcome and an indicator of mental well-being [1]. The relationship between mindfulness and mental health has been increasingly recognized [2,3]. Reliable and valid measures of mindfulness are important to facilitate research, assessment, and outcome evaluation. The Mindful Attention Awareness Scale (MAAS) [4] and the Five Facet Mindfulness Questionnaire (FFMQ) [5] are two commonly used measures of mindfulness. However, the MAAS has been increasingly criticized for several reasons. For example, its negative wordings make it more like a measure of dissociation, which has been proposed to be the opposite of mindfulness [6]. Moreover, the MAAS focuses heavily on the attentional aspect of mindfulness, and it does not measure some other important components, such as acceptance [7]. In contrast, the FFMQ is a well-recognized multidimensional measure of mindfulness, and its 39-item version has been validated across cultures [8]. Despite widespread use of the FFMQ-15, evidence among mental health service users and Chinese-speaking populations remains limited, leaving uncertainty about its validity in these contexts. Its length, however, limits its practicality for large-scale surveys or time-constrained settings, prompting the development of the 15-item FFMQ-15 to reduce participant burden while retaining psychometric robustness among non-clinical samples [9,10].

In this study, we further evaluated the psychometric properties of the FFMQ-15 in two samples of mental health service users with different cultural and linguistic backgrounds for the following reasons. The objectives of this study are (1) to evaluate the reliability and validity of the FFMQ-15 among mental health service users. (2) To assess the validity of a newly translated Chinese version of the FFMQ-15, given the limited evidence available for this population. (3) To examine measurement invariance across English- and Chinese-speaking groups to determine whether the scale functions equivalently across languages and cultures. First, although mental health service users exhibit lower levels of mindfulness [11], very few studies have validated the FFMQ-15 in this population. As mental health service users often have different struggles, such as challenges in recognizing or accepting emotions, further research is needed to confirm the validity of the FFMQ-15 as a measure of mindfulness in this population. Second, to our knowledge, the FFMQ-15 has been used in other culture [12], mindfulness facets may function differently in clinical populations because attentional awareness does not always translate into acceptance-related processes, particularly among individuals with emotional regulation difficulties. But evidence of its validity in Chinese populations is limited, as it has only been tested in a few studies, including a recent study with early adolescents [13]. Third, cross-cultural validation, including measurement invariance across English- and Chinese-speaking populations, requires additional empirical support to ensure comparable score interpretation. This study addresses these gaps by examining the reliability, validity, and cross-cultural applicability of the FFMQ-15 in two convenience samples of mental health service users, one English-speaking and one Chinese-speaking.

## 2. Research Questions

- Does the FFMQ-15 demonstrate adequate reliability?
- Does the five-factor model hold across groups?
- Is measurement invariance supported?

## 3. Methods

### 3.1. Participants

This study analyzed data from a cross-cultural online survey study, which obtained ethical approval at the Haliç University. In 2025, our team recruited potential participants using a snowballing technique and social media advertising. Recruitment was conducted via social media platforms (e.g., Facebook, Instagram, and local mental health forums), with snowball sampling extending through personal and professional networks. Recruitment strategies did not differ across regions, though reach may have varied by platform. Snowball sampling may introduce bias, as participants are recruited through existing networks. When recruiting potential participants, it was stated that the online survey aimed to examine predictors of mental health problems across cultures. To meet the inclusion criteria, participants should be aged 18 or above, be able to read and write English or Chinese, report receiving any kind of mental health services (e.g., counseling, social work, and/or psychiatric services) in the past 12 months. If participants reported that they were officially diagnosed with a reading disorder, dementia, or intellectual disabilities, they would be excluded. English- and Chinese-speaking participants were provided with the survey in their respective language. No incentives were provided for the participants.

The *English-speaking sample* consists of 115 English-speaking adult mental health service users. Most of them reported living in the United States (43.5%), Australia (34.8%) or Canada (18.3%). Their ages ranged from 18 to 69 years (M = 33.22; SD = 13.32). Most of them were female (72.2%). The heavy overrepresentation of female participants (>70%) is discussed as a potential source of bias, as gender imbalance may influence mindfulness reporting and limit generalizability. In the past 12 months, 56.5% reported seeing a psychiatrist, and 53.9% reported seeing a clinical psychologist.

*The Chinese-speaking sample* consists of 118 Chinese-speaking adult mental health service users. Most of them reported living in Taiwan (92.4%) or Hong Kong (7.6%). Their ages ranged from 18 to 58 years (M = 28.99; SD = 9.20); 80.5% were female. In the past 12 months, 74.6% reported seeing a psychiatrist, and 49.2% reported seeing a clinical psychologist.

### 3.2. Measure

Participants completed the FFMQ-15 in their respective languages. The FFMQ-15 is a shortened version of the original 39-item FFMQ, and it was found to be reliable and valid in several languages, including English [12] and Spanish [14]. In the present study, we used a collaborative approach [15,16,17] to translate the FFMQ-15 into Chinese. Principal component analysis (PCA) was selected for EFA to maximize explained variance, though we acknowledge debates in psychometrics regarding PCA versus common factor analysis. In particular, two translators (a nursing student and a social worker) first independently translated the FFMQ-15 into Chinese. The two Chinese versions were then compared, reviewed, and finalized in a panel of experts, including three PhD-level mental health researchers who have published peer-reviewed papers on mindfulness. Response formats and scoring procedures were identical across English and Chinese versions to ensure comparability. The use of the collaborative approach was to ensure equivalence in meaning and concepts instead of the literal equivalence across languages [15]. Emphasis was put on semantic translation. The panel of experts verified the face validity of the Chinese version of the FFMQ-15.

### 3.3. Data Analysis

Prior to all analyses, data quality was rigorously ensured. Incomplete responses were excluded from the dataset. Missing data were handled through listwise deletion. Outliers were identified and removed using standardized scores; cases with absolute z-scores exceeding ±3.3 on any item were excluded. The exclusion criterion of z > 3.3 led to the removal of 6 participants (2.6% of the sample). Sensitivity checks indicated that excluding these cases did not materially alter factor loadings or the overall structure. Outliers were excluded using ±3.3 z-scores, a conservative threshold to minimize distortion of factor distributions. Exploratory factor analysis (EFA) was conducted on the combined sample (N = 233) to identify the underlying factor structure of the 15-item Five Facet Mindfulness Questionnaire (FFMQ-15) and to inform potential structural dimensions of the observed variables. To perform the factor analysis, the Kaiser–Meyer–Olkin (KMO) test (>0.07) and Bartlett’s test of sphericity were used. PCA with varimax rotation was used to maximize explained variance; although debated in psychometrics, PCA was selected for its efficiency in data reduction, and results aligned with theoretical expectations.

Model fit was assessed using multiple goodness-of-fit indices: χ^2^ (preferably non-significant, *p* > 0.05, though noted to be sensitive to sample size), Comparative Fit Index (CFI) ≥ 0.90, Tucker–Lewis Index (TLI) ≥ 0.90, Root Mean Square Error of Approximation (RMSEA) ≤ 0.08, and Standardized Root Mean Square Residual (SRMR) ≤ 0.08 [18,19]. Subsequently, multi-group CFA was employed to test the measurement invariance of the five-factor model across the Chinese-speaking and English-speaking samples. Model invariance was examined sequentially, each with around 100 participants: (a) configural invariance (equivalent factor structure), (b) metric invariance (equivalent factor loadings), (c) scalar invariance (equivalent item intercepts), and (d) strict invariance (equivalent residual variances). Analyses were performed using R with the “lavaan” package. In addition to the lavaan package, analyses used psych and sem Tools packages in R to compute reliability indices and invariance testing as shown in Appendix B.

## 4. Results

The Exploratory Factor Analysis (EFA) yielded a Kaiser-Meyer-Olkin (KMO) measure of sampling adequacy of 0.774, indicating adequate sampling for factor analysis [20]. Bartlett’s Test of Sphericity was significant (χ^2^(105) = 1155.64, *p* < 0.001), confirming that the correlation matrix was suitable for factor analysis. Using PCA with varimax rotation, five components with eigenvalues greater than 1 were extracted, collectively accounting for 68.32% of the total variance. The first component explained 16.28% of the variance after rotation, followed by the second (13.79%), third (13.26%), fourth (12.81%), and fifth (12.18%) components. These results support a five-factor structure for the FFMQ-15, consistent with its theoretical framework, and all five subscales showed adequate to satisfying Cronbach’s Alpha and McDonald’s Omega values. Composite reliability (CR) values ranged from 0.685 to 0.800, indicating adequate to good internal consistency across facets. Internal consistency was supported, as alpha and omega values decreased when items were deleted, further confirming reliability. However, the Observing facet (CR = 0.689) showed slightly lower reliability, possibly due to its lower factor loadings (e.g., 0.520 for FFMQ1). All subscales showed a reduction in alpha and omega values of an item if deleted. Please refer to Table 1 for the EFA factor structure summary.

Confirmatory factor analysis (CFA) was conducted using maximum likelihood estimation (ML) to evaluate the five-factor structure of the FFMQ-15 in a combined sample of 233 English- and Chinese-speaking mental health service users. The model demonstrated acceptable fit: χ^2^/df = 159.50/80 = 1.99, *p* < 0.001; CFI = 0.927; TLI = 0.904; RMSEA = 0.065 (90% CI [0.050, 0.080]); SRMR = 0.060, BIC = 10,843.067, meeting established thresholds [18]. It should be noted that each language group included ~115 participants, which is at the lower bound for stable multi-group CFA with five latent factors. Some fit indices, such as TLI = 0.881 in the Chinese sample, fell slightly below conventional thresholds [18], warranting cautious interpretation. All factors were significant (*p* < 0.001), ranging from 0.520 to 0.865 (standardized), supporting the construct validity of the five facets: Observing (OB), Describing (DES), Acting with Awareness (ACT), Non-Judging (NONJ), and Non-Reactivity (NONR). Composite reliability (CR) values ranged from 0.685 to 0.800, indicating adequate to good reliability, while average variance extracted (AVE) values ranged from 0.382 to 0.654, with most constructs meeting or approaching the 0.50 threshold for convergent validity [21]. Moreover, the inter-factor correlations were generally significant (*p* < 0.05), except for OB with ACT, NONJ, and NONR, and ACT with NONR. Please refer to Table 2 for the factor loadings and reliability index summary. The Chinese sample, in particular, demonstrates acceptable fit even with a limited sample size, with χ^2^(80) = 120.677, χ^2^/df = 1.51, *p* = 0.002; CFI = 0.909; TLI = 0.881; RMSEA = 0.066 (90% CI [0.040, 0.089]); SRMR = 0.078; BIC = 5676.383. All factor loadings were statistically significant (*p* < 0.001), supporting the construct validity of the hypothesized facets. The full factor loading table with residuals is represented in Appendix A.

**Figure 1:**
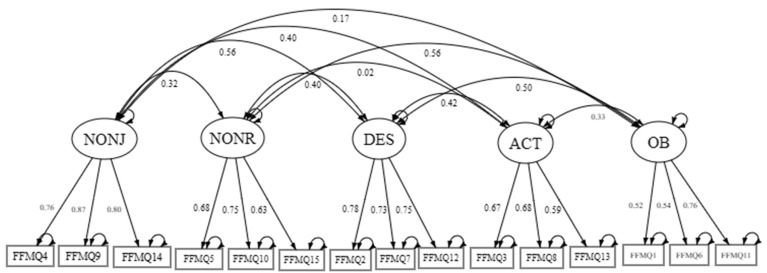
CFA Diagram. The Confirmatory Factor Analysis Diagram of 15-item Five Facet Mindfulness Questionnaire. Note: Observing (OB), Describing (DES), Acting with Awareness (ACT), Non-Judging (NONJ), and Non-Reactivity (NONR).

Confirmatory factor analysis (CFA) was performed on the combined sample (N = 233) to evaluate the fit of the theoretically derived five-factor structure of the FFMQ-15. A CFA diagram (Figure 1) is provided to visually illustrate the five-factor structure.

Measurement model invariance of the FFMQ-15 across English-speaking and Chinese-speaking mental health service user samples was examined. However, the scalar invariance model, constraining both loadings and intercepts, yielded a poorer fit, with a significant chi-squared difference (Δχ^2^(10) = 67.553, *p* < 0.001) and ΔCFI = −0.049, indicating a lack of scalar invariance. These results indicate that the FFMQ-15’s factor structure and loadings are consistent across the two samples, enabling meaningful comparisons of factor variances and covariances, but differences in intercepts prevent direct mean comparisons.

**Figure 2:**
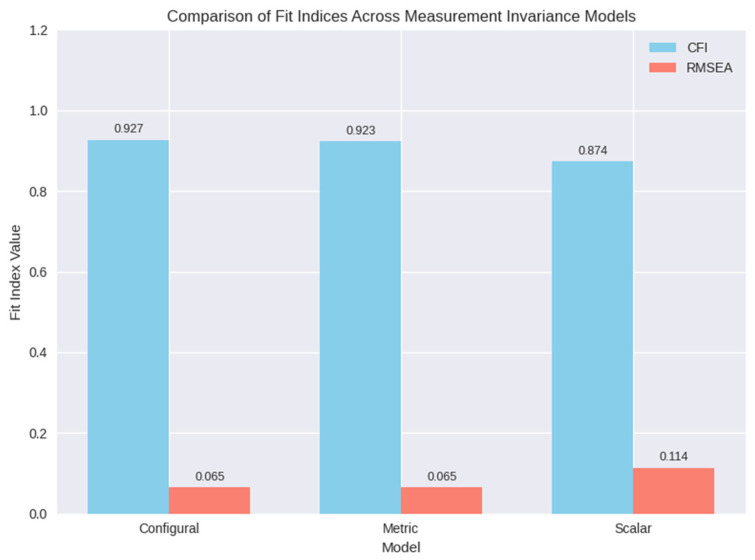
Invariance Model Comparisons.

In addition, a visual representation of invariance model comparisons is shown in Figure 2 below.

## 5. Discussion

The current paper evaluated the psychometric properties of the 15-item Five Facet Mindfulness Questionnaire (FFMQ-15) in a combined sample of English-speaking (n = 118) and Chinese-speaking (n = 115) mental health service users. The modest sample size (115 per group) represents a limitation for multi-group CFA, as smaller samples may reduce stability of parameter estimates and increase sensitivity of fit indices. The slightly lower TLI in the Chinese sample underscores this limitation. The EFA and CFA showed literature-aligned results in supporting the 5-factor structure model, demonstrating the consistency of the measurement model among clinical patients and in a cross-cultural context. Yet, there are a few points that are worth discussing.

The absence of scalar invariance implies that researchers cannot assume equivalence of mean scores across English and Chinese samples. Future cross-cultural comparisons should rely on latent variance and covariance rather than raw scale means. The observing facet’s lower AVE suggests it may be less cohesive in mental health service users, and with non-significant correlations between Observing and other facets (ACT, NONJ, NONR), which is consistent with previous findings that Observing may function differently in clinical versus non-clinical samples [22]. Comparison with previous Chinese mindfulness literature [13,22] suggests that cultural-conceptual considerations, such as emotional restraint and relational self-construal, may explain differences in Observing facet functioning. These findings may reflect cross-cultural differences in emotional expression and self-awareness. In collectivist contexts, such as Chinese-speaking populations, cultural norms emphasize emotional restraint and relational self-construal, which may reduce the salience of observing internal states [23]. Consequently, the Observing facet may capture attentional awareness without necessarily linking to acceptance processes. Prior cross-cultural validation studies confirm that Observing functions inconsistently across countries, suggesting cultural norms shape item meaning [8]. Moreover, cultural differences in emotional regulation strategies, such as suppression or reappraisal, may moderate mindfulness outcomes and explain weaker correlations between Observing and other facets in clinical samples [24]. Network analyses further show that the associations between mindfulness facets and personality traits vary by cultural context, with Observing aligning more strongly with openness in Western samples than in collectivist contexts [25]. Taken together, these perspectives highlight the importance of culturally sensitive adaptations when applying the FFMQ-15 across diverse populations. This suggests that attentional awareness might be less integrated with acceptance-related processes among individuals receiving mental health services. In contrast, Peters et al. reported varying levels of internal consistency across the mindfulness facets, with non-reactivity consistently exhibiting lower reliability [26]. Non-reactivity consistently shows lower reliability [22]. Supplementary sensitivity checks are in Appendix B. While attentional awareness is a foundational element of mindfulness practice, translating into non-judgmental acceptance is neither automatic nor universally applicable.

Measurement invariance testing indicated configural and metric invariance across language groups, enabling valid comparisons of factor loadings and structural relationships. However, the lack of scalar invariance implies caution when interpreting mean differences between English- and Chinese-speaking samples. This pattern mirrors cross-cultural research showing variability in mindfulness conceptualization [8], where cultural norms around emotional expression and self-awareness may influence item interpretation.

Compared to prior studies in general populations [12,14], the current findings extend evidence to mental health service users—a group often characterized by lower mindfulness levels [11]. The acceptable psychometric properties observed here underscore the FFMQ-15’s utility in clinical contexts, though facet-specific nuances warrant further investigation.

## 6. Practical Implications and Limitations

The present findings contribute to the nascent body of research examining the psychometric properties of the 15-item Five Facet Mindfulness Questionnaire (FFMQ-15) in mental health service users, particularly within Chinese-speaking populations. Building on prior validation studies in clinical and cross-cultural contexts [10], this study provides further evidence of the instrument’s utility in diverse linguistic groups. The lack of scalar invariance has important implications: latent mean comparisons across English- and Chinese-speaking groups should be interpreted with caution, and future research may explore culturally adapted items to improve invariance. The FFMQ-15 exhibited robust psychometric properties overall, rendering it a reliable and concise tool for assessing mindfulness facets in both English- and Chinese-speaking mental health service users. The establishment of configural and metric invariance across language groups supports the cross-cultural comparability of factor structures and loadings, facilitating meaningful comparisons of structural relationships. Cultural adaptation of specific items may be necessary to improve scalar invariance and ensure valid cross-group mean comparisons. Notably, the validated Chinese version addresses a salient gap in the literature, enabling culturally sensitive mindfulness assessment among Chinese-speaking clinical populations.

Several limitations warrant consideration. The total sample size (N = 233), divided across two language groups (n ≈ 115–118 per group), was modest and approached the lower threshold for multi-group confirmatory factor analysis, which may have constrained statistical power and the stability of parameter estimates. Convenience sampling further limits the generalizability of findings, as the sample may not fully represent the broader population of mental health service users. Both the configural and metric invariance model showed adequate fit, and the chi-squared difference test between configural and metric models was non-significant (Δχ^2^(10) = 15.007, *p* = 0.1318), and changes in fit indices were minimal (ΔCFI = −0.004, ΔRMSEA = 0.000), supporting full metric invariance [27]. Additionally, the overrepresentation of female participants (>70% in both groups) introduces potential gender-related biases and restricts applicability to male service users. Future research should test alternative short forms of mindfulness measures and incorporate qualitative methods to explore cultural interpretations of items.

## 7. Conclusions

Despite these limitations, the FFMQ-15 demonstrates considerable promise as a psychometrically sound and practical instrument for mindfulness assessment in multilingual clinical and research contexts. While configural and metric invariance support cross-cultural comparability, the lack of scalar invariance requires caution when comparing latent means. Future studies with larger, more diverse samples are recommended to strengthen measurement invariance evidence and enhance the instrument’s cross-cultural validity. Overall, the FFMQ-15 shows strong psychometric properties for measuring mindfulness in English- and Chinese-speaking mental health populations, supporting its value in clinical research and practice. The validated Chinese version of the FFMQ-15 provides a foundation for regional clinical research and supports culturally sensitive mindfulness assessment in Chinese-speaking populations.

## Figures and Tables

**Table 1:** Exploratory Factor Analysis Results.

Constructs	Measurements	Factor Loading				
		1	2	3	4	5
NONJ	FFMQ4	0.796				
	FFMQ9	0.841				
	FFMQ14	0.853				
NONR	FFMQ5		0.682			
	FFMQ10		0.824			
	FFMQ15		0.800			
DES	FFMQ2			0.830		
	FFMQ7			0.775		
	FFMQ12			0.732		
ACT	FFMQ3				0.793	
	FFMQ8				0.766	
	FFMQ13				0.692	
OB	FFMQ1					0.703
	FFMQ6					0.667
	FFMQ11					0.748
Eigenvalue		4.002	2.546	1.568	1.083	1.049
% of Variance Explained		16.277	13.794	13.259	12.806	12.184
Cronbach’s Alpha		0.848	0.797	0.729	0.682	0.616
McDonald’s Omega		0.850	0.735	0.799	0.683	0.646

Notes: Extraction Method: Principal Component Analysis. Rotation Method: Varimax with Kaiser Normalization. Total variance explained = 68.32%. KMO = 0.774, Bartlett’s Test of Sphericity = χ^2^(105) = 1155.64, *p* < 0.001. No items require reverse coding. Observing (OB), Describing (DES), Acting with Awareness (ACT), Non-Judging (NONJ), and Non-Reactivity (NONR).

**Table 2:** Confirmatory Factor Analysis Model Fit Statistics.

Construct	Measurement	Factor Loading	AVE	CR
**NONJ** (Non-Judging)	FFMQ4	0.755	0.654	0.741
	FFMQ9	0.865		
	FFMQ14	0.802		
**NONR** (Non-Reactivity)	FFMQ5	0.681	0.478	0.732
	FFMQ10	0.754		
	FFMQ15	0.634		
**DES** (Describing)	FFMQ2	0.784	0.570	0.800
	FFMQ7	0.732		
	FFMQ12	0.749		
**ACT** (Acting with Awareness)	FFMQ3	0.669	0.420	0.685
	FFMQ8	0.679		
	FFMQ13	0.594		
**OB** (Observing)	FFMQ1	0.520	0.382	0.689
	FFMQ6	0.544		
	FFMQ11	0.762		

Notes: All factor loadings are standardized (Std.all) and significant (*p* < 0.001). Average Variance Extracted (AVE) and Composite Reliability (CR) are reported at the construct level. Constructs: Observing (OB), Describing (DES), Acting with Awareness (ACT), Non-Judging (NONJ), and Non-Reactivity (NONR).

## Data Availability

The data presented in this study are available on request from the corresponding author. The data are not publicly available due to privacy and ethical restrictions.

## Appendix A. Standardized Factor Loadings, Residual Variances, R^2^ and Modification Indices for FFMQ Items

**Table A1:** Standardized Factor Loadings for the FFMQ-15 (Combined Sample).

Construct	Item	Standardized Loading	Residual Variance	R^2^
NONJ	FFMQ4	0.755	0.430	0.570
	FFMQ9	0.865	0.251	0.749
	FFMQ14	0.802	0.357	0.643
NONR	FFMQ5	0.681	0.536	0.464
	FFMQ10	0.754	0.432	0.568
	FFMQ15	0.634	0.598	0.402
DES	FFMQ2	0.784	0.386	0.614
	FFMQ7	0.732	0.464	0.536
	FFMQ12	0.749	0.439	0.561
ACT	FFMQ3	0.669	0.553	0.447
	FFMQ8	0.679	0.539	0.461
	FFMQ13	0.594	0.647	0.353
OB	FFMQ1	0.520	0.730	0.270
	FFMQ6	0.544	0.704	0.296
	FFMQ11	0.762	0.419	0.581

**Table A2:** Largest Modification Indices (>10).

Parameter	MI Value	Expected Change
OB=~ FFMQ2	16.32	+0.70
OB=~ FFMQ7	12.78	−0.68
NONJ=~ FFMQ2	17.15	−0.61
NONR=~ FFMQ2	13.48	+0.68
NONR=~ FFMQ7	11.49	−0.69

## Appendix B. R

# Load required packages
library (lavaan)
library (semTools)
library (psych)

# Define CFA model for FFMQ-15
ffmq_model <- ‘
NONJ =~ FFMQ4 + FFMQ9 + FFMQ14
NONR =~ FFMQ5 + FFMQ10 + FFMQ15
DES =~ FFMQ2 + FFMQ7 + FFMQ12
ACT =~ FFMQ3 + FFMQ8 + FFMQ13
OB =~ FFMQ1 + FFMQ6 + FFMQ11
‘
# Fit the model
fit ← cfa (ffmq_model, data = your_data, estimator = “ML”)

# --- Factor Loadings ---
summary (fit, standardized = TRUE, fit.measures = TRUE)

# --- Residuals ---
residuals (fit, type = “standardized”)

# --- Modification Indices ---
modindices (fit, sort = TRUE, minimum.value = 10)
